# HAuCl_4_, Putative General Aquaporins Blocker, Reduces Platelet Spreading, Filopodia Formation, Procoagulant Response, and Thrombus Formation Under Flow

**DOI:** 10.3389/fphys.2020.01025

**Published:** 2020-08-21

**Authors:** Tomasz Misztal, Agata Golaszewska, Justyna Branska-Januszewska, Natalia Marcinczyk, Ewa Chabielska, Marian Tomasiak, Tomasz Rusak

**Affiliations:** ^1^Department of Physical Chemistry, Medical University of Bialystok, Bialystok, Poland; ^2^Department of Biology, Medical University of Bialystok, Bialystok, Poland; ^3^Department of Biopharmacy, Medical University of Bialystok, Bialystok, Poland

**Keywords:** platelets, aquaporins, thrombus formation, mouse model of thrombosis, procoagulant activities, phosphatidylserine exposure

## Abstract

**Background**: Recent studies indicate that aquaporin (AQP) water channels have a regulatory function in human platelet secretion and in procoagulant response of murine platelets. However, the engagement of AQPs in morphological changes, procoagulant response, and thrombus formation in human blood has never been investigated.

**Methods**: Confocal microscopy was used to study platelet spreading, filopodia formation, ballooning, and thrombus formation under flow. Flow cytometry was utilized to assess platelet phosphatidylserine (PS) exposure and microparticles shedding. Kinetics of clot formation *in vitro* was evaluated by thromboelastometry. Mouse model of ferric chloride (III) (FeCl_3_)-induced thrombosis was used to investigate thrombus formation *in vivo*.

**Results**: We found that chloroauric(III) acid (HAuCl_4_), a classical AQP inhibitor (10–100 μM), reduced spreading of human platelets on collagen-coated surfaces and inhibited filopodia formation in a fluid phase. Under flow conditions, HAuCl_4_ (100 μM) attenuated thrombi growth on collagen, platelet secretion, and PS exposure. Thrombus formation was restored by the addition of exogenous adenosine diphosphate (ADP). Collagen-evoked platelet procoagulant response (evaluated as PS exposure, shedding of microparticles, platelet-dependent thrombin generation, and membrane ballooning) was distinctly reduced by HAuCl_4_ (25–200 μM), as well as the dynamics of clot formation. In mouse model of thrombosis, reduction of surface of PS-positive cells within thrombus was observed in the presence of HAuCl_4_ (1–10 mg/kg).

**Conclusion**: These results suggest that in human platelets AQPs are crucial for agonist-evoked morphological changes, thrombus formation under flow, and in development of procoagulant response. Antithrombotic effect *in vivo* suggests that nontoxic inhibitors of AQPs may be considered as potential candidates for a novel class of antiplatelet drugs.

## Introduction

Aquaporin (AQP) water channels are members of a large, evolutionarily conserved family of integral membrane proteins that mediate rapid and selective transport of water and small solutes (e.g., glycerol) across cell and organelle membranes in response to osmotic gradient generated by ion transporters and exchangers (Agre et al., 2002; Kato et al., 2006; Hub and de Groot, 2008; Carbrey and Agre, 2009; Verkman, 2012).

Topologically, AQPs are homotetramers forming four channels within the membrane, where each of them acts as independent water transductor (Sui et al., 2001; Khalili-Araghi et al., 2009; Walz et al., 2009). Central region of each monomer contains a highly conserved short hydrophobic region with the characteristic asparagine-proline-alanine motifs (NPA boxes) flanking a narrowing (~2.8 Å in diameter for AQP1) allowing a single-file water transport (Sui et al., 2001; Verkman, 2012).

To date, 13 members of the AQP family (labeled AQP0–AQP12) have been described in various mammalian cells (Agre et al., 2002; Benga, 2012; Ishibashi et al., 2014). Recently AQPs, next to their “classical” role in cell adaptation to changes in osmotic pressure (Agre et al., 2002; Verkman, 2014), have been connected with formation of filopodia and lamellipodia, and with swelling of secretory vesicles, which is believed to be a crucial step in the exocytosis process (Loitto et al., 2007; Sugiya et al., 2008; Louchami et al., 2012; von Bülow et al., 2012; Karlsson et al., 2013; Misztal et al., 2018).

AQPs have been found in human, rat, and murine platelets (Lee et al., 2012; Goubau et al., 2013; Agbani et al., 2018; Misztal et al., 2018). In rats, AQP6 has been proposed to be associated with platelet volume regulation (Lee et al., 2012). In mice, deletion of AQP1 has been shown to be related with a reduction of platelet procoagulant response and thrombus formation *in vivo* but had minimal effect on platelet secretion and aggregation triggered by collagen *in vitro* (Agbani et al., 2018). In humans, homozygosity in the AQP7 gene (G264V mutation) has been connected with the reduction of adrenaline-evoked platelet aggregation and secretion from dense granules triggered by collagen or adenosine diphosphate (ADP; Goubau et al., 2013). Our recent study, employing detection with polyclonal antibodies, suggests the possible presence of 12 isoforms of AQPs in human platelets localized within plasma membrane and secretory granules (Misztal et al., 2018). Next, we showed that blockage of AQPs in platelets resulted not only in a strong reduction of osmolality‐ or agonist-evoked platelet swelling but also in a complete inhibition of dense granules secretion, and in a very strong reduction of release reaction from alpha granules and lysosomes, in platelet, triggered by a broad panel of physiological and pharmacological stimuli. Yet, the knowledge of the involvement of AQPs in human platelet responses is still fragmentary. Particularly, there is no information about impact of AQPs’ inhibition on thrombus formation under flow conditions and platelet procoagulant response in human blood.

Since numerous isoforms of AQPs are expected to be present in human platelets, studies with genetic knockouts, restricted to deletion of one particular AQP isoform, may thus be affected by compensatory effect of other AQPs present in platelets. Therefore, to better understand the role of AQPs in platelet physiology and to evaluate platelet AQPs as potential targets for future antiplatelet therapeutics, studies with general inhibition of platelet AQPs are prospective.

The great number of compounds has been tested as potential APQs’ blockers but finding an isoform-specific inhibitor is still a challenging effort (Verkman et al., 2014; Tradtrantip et al., 2017). The most extensively used nonspecific AQPs’ inhibitor – HgCl_2_, is highly toxic and its general inhibitory properties has been questioned since activation of AQP6 by HgCl_2_ has also been observed (Lee et al., 2012). What is more, mercurials (including HgCl_2_) have been found to notably increase human platelet procoagulant activity at concentrations even lower than those routinely used to block AQPs (Goodwin et al., 1995), which makes them unsuitable tools to study antiplatelet effect of AQPs inhibition. Studies performed on various cell types suggest that gold-containing compounds are promising candidates for general inhibitors of AQPs, with comparable inhibitory potential to HgCl_2_ but without side-effects and direct toxicity toward platelets (Niemietz and Tyerman, 2002; Yang et al., 2006; Martins et al., 2013; Misztal et al., 2018). For instance, chloroauric(III) acid [HAuCl_4_, referred to here as “Au(III)”] has been shown to strongly inhibit water permeability of peribacteroid membrane from soybean, murine and human red blood cells, and human platelets (Niemietz and Tyerman, 2002; Yang et al., 2006; Misztal et al., 2018). Notably, in platelets, water uptake induced by hypotonia or agonists, as well as secretory granules swelling and platelet secretion have been strongly reduced (Misztal et al., 2018). Of importance, the toxicity of Au(III) toward human erythrocytes (evaluated as hemoglobin release) and platelets (considered as plasma membrane integrity and total thiol groups content) was negligible at concentrations notably reducing swelling and secretion (Misztal et al., 2018). Inhibition of water channels by Au^3+^-containing compounds is believed to occur *via* steric hindrance of AQP pore provided by interaction of gold ion with the -SH group of the specific cysteine residue (Cys-191) protruding into the water channel (Martins et al., 2013; Esteva-Font et al., 2016). Additionally, Au^3+^ with a diameter of ion of ~2.7 Å matches the predicted typical AQP pore diameter (~2.8 Å; Niemietz and Tyerman, 2002). This combination of reactivity and size most likely constitutes the basis of its inhibitory effect. Due to above reasons, in this study we used Au(III) as a putative general inhibitor of AQPs.

Consequently, this study was designed to evaluate the impact of general blockage of AQPs on platelet morphological changes (filopodia formation and spreading) upon activation, thrombus formation under flow, and procoagulant response using human blood *in vitro* and mouse intravital experimental models.

## Materials and Methods

### Materials

Cruz Fluor 405-phalloidin and Alexa Fluor 488-conjugated anti-α-tubulin antibody were purchased from Santa Cruz Biotechnology. Alexa Fluor 647-conjugated annexin V (ANX V), Alexa Fluor 488-conjugated anti-human CD63 antibody, Fura 2-AM, and DiOC6(3) {3-octadecyl-2-[3-(3-octadecyl-2(3H)-benzoxazolylidene)-1-propenyl]-, perchlorate}, were from Thermo Fisher Scientific (Life Technologies). Mepacrine and HAuCl_4_ tetradydrate were purchased from Sigma. Fibrillar collagen type I (Horm suspension) for adhesion was purchased from Nycomed. Fluorescein isothiocyanate (FITC)-conjugated ANX V, phycoerythrin (PE)-conjugated mouse anti-human CD41a antibody, FITC-conjugated mouse anti-human CD62P (P-selectin) antibody, and FITC-conjugated PAC-1 antibody were purchased from Becton Dickinson. Tissue factor (TF; Innovin) was purchased from Dade Behring. Tirofiban (Aggrastat) were from Merck Sharp & Dohme Idea Inc. Ketamine and xylazine were purchased from Biowet. Other chemicals were purchased from Sigma. HAuCl_4_ tetrahydrate was dissolved in deionized water and stock solutions (1 M conc.) were stored at −80°C. Working solutions were prepared by diluting of stock solution with sterile phosphate-buffered saline (PBS) and kept at −20°C for no longer than 3 weeks. Attention was taken to do not exceed 0.25% (v/v) of HAuCl_4_ solution in a sample (final concentration). Such additions did not evoke any noticeable changes in samples’ pH.

### Human Blood Collection and Platelet Preparation

Blood was collected from healthy volunteers into 10 ml polypropylene tubes containing 1 ml of 130 mM trisodium citrate. All procedures were conducted in accordance with the Declaration of Helsinki, and the study was approved by the local Bioethics Committee on human research (R-I-002/224/2015 and R-I-002/339/2017). Platelet-rich plasma (PRP) and washed platelets suspensions were prepared as described in (Misztal et al., 2015).

### Animals

Ferric chloride (III) (FeCl_3_)–induced thrombosis was performed using *male wild type C57BL/6* J mice. Mice weighted 20–23 g. All animals were purchased from Center for Experimental Medicine in Bialystok. After the experiments animals were killed by cervical dislocation. Procedures involving animals and their care were conducted in conformity with the institutional guidelines that are in compliance with national and international laws and Guidelines for the Use of Animals in Biomedical Research. All the procedures involving the animals and their care were approved by the Local Ethical Committee on Animal Testing at the Medical University of Bialystok (Permit number: 93/2018).

### Estimation of Platelet Filopodia Formation in Fluid Phase

Samples of standardized PRP (2 × 10^8^ cells/ml) supplemented with tirofiban (250 ng/ml), were stained using DiOC6(3) (2 μM final conc., 20 min at RT) followed by incubation in the cuvette of an aggregometer with or without (control) HAuCl_4_, (100 μM final conc.) for 2 min at 37°C with stirring. Then, platelets were stimulated with the threshold concentrations of collagen (typically 15 μg/ml) or ADP (10 μM), and the trace reflecting shape change was monitored. At the point of maximal shape change (decrease in the baseline of light transmittance), samples were fixed by the addition of an equal volume of 25°C-warm 4% paraformaldehyde in PBS. After 30 min of fixation at RT, platelets were pelleted by centrifugation (2,200 × *g*, 2 min) and resuspended in a fresh PBS. Aliquots (25 μl) of platelets were transferred into microchamber slides and observed under confocal microscope [Nikon ECLIPSE Ti/C1 Plus (Ex 488 nm/Em 515/30 nm, ×100/1.4 oil immersion objective)]. At least 10 pictures of different areas of each cell suspension were made.

### Quantification of Platelet Adhesion and Spreading on Collagen

Adhesion of platelets to collagen-coated surfaces was performed under static conditions using glass microscopic slides covered by collagen (Horm preparation, 50 μg/ml). Aliquots (20 μl) of washed platelets preincubated for 2 min at 37°C with or without HAuCl_4_, added to the final concentrations as indicated, were supplemented with MgCl_2_ and CaCl_2_ (3 mM final conc. of both), transferred onto collagen-coated slides and covered with coverslips. After incubation for 30 min at 37°C, platelets were visualized using brightfield microscopy. Number of adhered platelets and covered area were quantified using ImageJ free software (National Institutes of Health image software^1^).

### Visualization of Actin and Tubulin Dynamics in Thrombin-Stimulated Platelets

Gel-filtered platelets (2 × 10^8^/ml) were incubated for 2 min at 37°C without or with HAuCl_4_ (100 μM) followed by the addition of thrombin (Thr, 5 nM). Control samples were without any additions (unstim). After 1 min, platelets were fixed (with 25°C-warm paraformaldehyde in PBS, 2% final conc.) saponized (0.04% final conc.), and incubated overnight with Cruz Fluor 405-phalloidin conjugate (for actin staining, 1:100 dilution) and anti-α tubulin antibody conjugated with Alexa Fluor 488 (1:250 dilution) and imaged using the same gain setting after which fluorescence was read for areas comprising the same number of cells by confocal microscope.

### Thrombus Formation Under Flow

To determine thrombus formation under arterial shear rate (1,000 s^−1^), we used parallel-plate flow chamber (de Witt et al., 2014; with chamber dimensions: height – 50 μm, width – 3 mm, and length – 30 mm). Thrombi formed on collagen (type I) were further analyzed toward their heights, surface coverage, phosphatidylserine (PS) exposure and procoagulant index, and platelet secretion markers (Mattheij et al., 2016; Misztal et al., 2019) using fluorescent microscope with a confocal module. In some experiments, whole blood samples were preincubated with DiOC6(3) (1 μM) to visualize platelet membranes (for further measurements of thrombi heights and surface coverage area) or with mepacrine (5 μM) to visualize not emptied dense granules within platelets. End-stage measurement was conducted using following conjugates: Alexa Fluor 647-conjugated ANX V to measure PS exposure and procoagulant index, and to estimate balloons number and diameters (1:200 dilution), Alexa Fluor 488-anti CD63, as a marker of dense granules secretion (1:50 dilution), and FITC-anti-P-selectin, as a marker of alpha granules secretion (1:20 dilution).

### Measurement of Coagulation Kinetics

For evaluating the coagulation kinetics, we used rotational thromboelastometry system (ROTEM; Luddington, 2005). We measured following parameters: clotting time (CT), alpha angle (α), and maximum clot firmness (MCF). Moreover, the following derivative illustrating the speed of clot initiation and propagation were analyzed: maximum velocity (MaxVel), time to obtain MaxVel, and area under curve (AUC). More detailed description of measured parameters is in Misztal et al. (2015). Coagulation was triggered by the addition of calcium chloride (“Ca^2+^”, 12 mM final conc.) or TF (Innovin, 140 ng/ml) with the presence of calcium chloride (“low TF”). All ROTEM measurements were performed as described in (Misztal et al., 2013).

### Measurement of Platelet Procoagulant Response

Measurements of PS exposure on the surface of collagen-activated platelets and shedding of platelet-derived microparticles (PMPs) by flow cytometry, and generation of thrombin by colorimetric assay were conducted as described previously in (Misztal et al., 2015).

### Ferric Chloride (III) Induced-Thrombosis in Mice

To thrombus induction, we used the procedure that was described in our previous study (Gromotowicz-Poplawska et al., 2019; Marcinczyk et al., 2020). In experiment, male wild type *C57BL/6*J mice were used. Wild type C57Bl/6J mice were anesthetized with ketamine and xylazine (120 mg/kg, *i.p.*, Ketamina 10%; 12.5 mg/kg, *i.p.*, Xylapan) after which 0.05 ml of HAuCl_4_ solution or 0.05 ml of PBS (VEH) was injected into left femoral vein and labeled annexin V (ANX V, Alexa Fluor 647 conjugate) was injected into the right femoral vein (2 mg/kg, 0.05 ml in PBS) 5 min before thrombosis induction. ANX V binds to PS exposed on the surface of irreversibly activated platelets (PS-positive platelets). To visualize vessel wall and PS-negative platelets, DiOC6(3) [0.1 mM in 0.05 ml of the mixture of DMSO and PBS (volume ratio 1:50)] was administrated with intramuscular injection 5 min before thrombosis induction. Then, midline laparotomy incision was made, and the mesentery of the ileum was pulled out of the abdomen and draped over a plastic mound. The volume of 0.1 μl of 20% water solution of FeCl_3_ was administrated topically at the mesenteric vein and immediately washed out with PBS. Topical application of the ferric chloride solution results in the diffusion of positively charged Fe(H_2_O)^3+^ ions and attraction to the vessel wall: the negatively charged erythrocytes and plasma proteins and then platelets undergo activation. Due to the hydrolysis, Fe(H_2_O)^3+^ precipitates to form Fe(OH)_3_ which flocculates platelet aggregates, red blood cells, and plasma proteins (Ciciliano et al., 2015). At the core of such evoked thrombus, platelets are activated irreversibly, which is reflected in the redistribution of PS from the inner leaflet to the outer surface of platelet plasma membrane. The mesentery vein was placed under the objective, and thrombus was identified. The mesentery was continuously perfused with 37°C-warmed PBS to prevent the vessels from drying. Alexa Fluor 647 dye was excited with 640 nm laser, and DiOC6(3) was excited by 488 nm laser (LaserStack 488 nm, LaserStack 640 nm, 3iL33, Intelligent Imaging Innovations, Inc., United States). The two color fluorescent pictures using confocal microscope were taken in one 2D focal plane corresponding with cross-section with the biggest area of platelets with attached ANX V (further referred as ANX V-positive platelets). The area of ANX V-positive platelets was then encircled and calculated with SlideBook 6.0 (Intelligent Imaging Innovation, Inc., Unites States). In all experiments Zeiss Axio Examiner Z.1 with Yokogawa CSU-X1 confocal scanning unit was used. All the experiments were performed under W Plan-Apochromat 20x/1.0 water immersion objective. In one mouse, one thrombus was induced.

### Clot Retraction Kinetics Measurements

The kinetics of clot retraction in whole blood was evaluated by optical method (Misztal et al., 2015). Pictures were taken for 1 h at 10 min intervals using a digital camera. Quantification of retraction was performed by assessment of the clot area by use of Motic Images Plus 2.0. Data were expressed as follows: percentage of retraction = [(area *t*_0_-area *t*)/area *t*_0_] × 100. Kinetics of clot retraction was characterized by calculating the rate constant of the retraction process (slope of the curve of retraction percentage vs. time; % × min^−1^). Because clot volume does change uniformly between approximately 10 and 50 min, for calculations of rate constant, retraction percentages at 20, 30, and 40 min were selected.

### Statistical Analysis

The data are shown as mean ± SEM or median (interquartile range) and analyzed using either the Student’s *t*-test (when the normality test passed) or the Mann–Whitney test (when the normality test failed). *p* < 0.05 was considered significant. All analyses were done in GraphPad Prism 5.

## Results

### Inhibitory Effect of Au(III) on Platelet Spreading and Filopodia Formation

**Figure 1:**
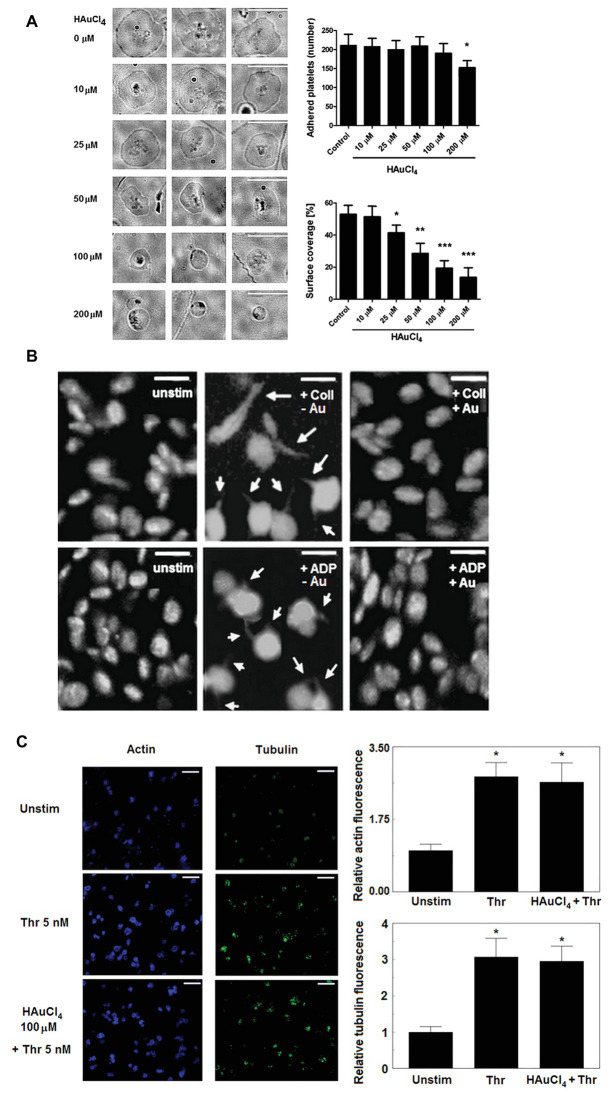
Effect of HAuCl_4_ on platelet adhesion, spreading, filopodia formation, and dynamics of actin and tubulin. **(A)** Washed platelets preincubated without (control) or with HAuCl_4_ (0–200 μM), were transferred onto collagen-coated glasses and visualized using brightfield microscopy (bar is 10 μm). A number of adhered platelets in a field of view of 0.01485 mm^2^ and surface coverage by adhered platelets (in %) are presented. Data (mean values ± S.D.) and representative images from four independent experiments are shown. **(B)** Platelet-rich plasma (PRP) samples, previously tirofiban-treated and stained with DiO, were preincubated with (+Au) or without (−Au) HAuCl_4_ (100 μM) and stimulated by collagen (Coll) or adenosine diphosphate (ADP) in an aggregometer cuvette. At the point of maximal recorded shape change, platelets were fixed, transferred into microchamber slides, and observed under confocal microscope. Unstimulated platelets (unstim) were used as a control. Magnifications of representative pictures from four independent experiments are presented (bar is 5 μm). Arrows indicate platelet membrane protrusions. Some adjustments in brightness and contrast were made to better accentuate thin, finger-like filopodia. **(C)** Content of polymerized actin and tubulin in unstimulated and thrombin-stimulated (Thr) platelets was assessed by means of confocal microscopy using fixed (post stimulation), saponized washed platelets labeled with Cruz Fluor 405-phalloidin (for F-actin staining) and anti-α-tubulin antibody conjugated with Alexa Fluor 488. At least 10 different areas from each sample were imaged using confocal microscope. Fluorescence was read for areas comprising the same number of cells. Representative images from one (out of three) experiment are presented. Bar is 10 μm, ^*^p < 0.05.

To test whether Au(III) may affect activation-dependent morphological changes of platelets, we examined its effect on lamellipodia and filopodia formation. Au(III) (10–200 μM) diminished platelet spreading and lamellipodia formation on fibrillar (type I) collagen-coated surfaces, in a dose-dependent manner (Figure 1A). Au(III), at concentration strongly reducing spreading (100 μM), markedly inhibited filopodia formation by collagen‐ or ADP-stimulated platelets in the fluid phase (Figure 1B).

This effect was not associated with Au(III) impact on total degree of actin and tubulin polymerization since Au(III) (100 μM) did not affect a rise in polymerized form of actin and tubulin following stimulation of platelets by thrombin (Figure 1C).

### Inhibitory Effect of Au(III) on the Formation of Thrombi, Phosphatidylserine Exposure, and Platelet Secretion Under Flow Conditions

**Figure 2:**
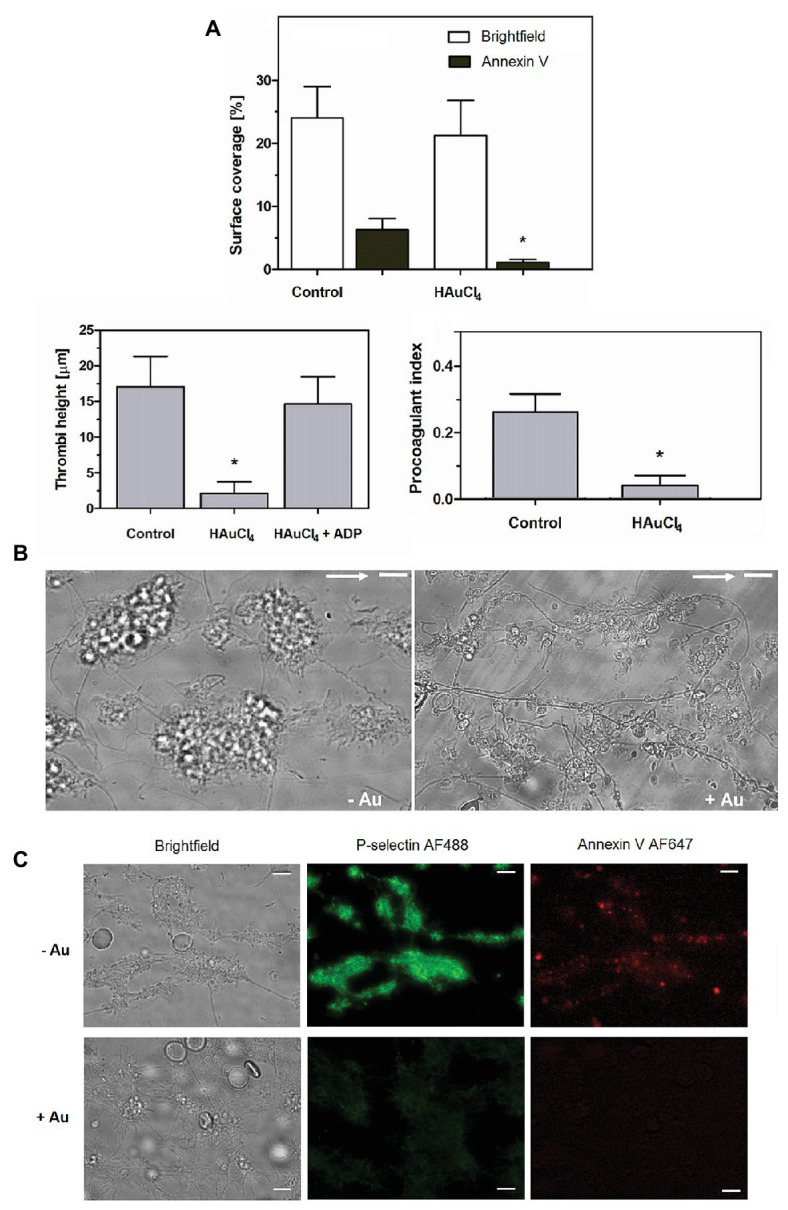
HAuCl_4_ inhibits GPVI-dependent thrombus growth, exposure of P-selectin and phosphatidylserine (PS) but not platelet adhesion to collagen under flow. Whole blood samples supplemented (+Au) with HAuCl_4_ (100 μM) were perfused over collagen at a shear rate of 1,000 s^−1^ followed by washing with buffer containing fluorescently labeled annexin V (ANX V; 1:200) and anti-P-selectin antibody (1:20). **(A)** Surface area coverage was calculated in a brightfield mode while thrombi heights were calculated from z-stack confocal sections. Samples of whole blood preincubated with ADP were perfused over monolayers composed of HAuCl_4_-treated platelets to evaluate the role of secretion in HAuCl_4_-evoked attenuation of thrombi formation. Procoagulant index values reflect relative number of PS-exposing platelets (*n* = 6). **(B)** Representative bright field images of thrombi developed in the absence (-Au) and in presence (+Au) of HAuCl_4_ from one experiment (out of six) are presented. Flow direction is shown by arrows. **(C)** Representative pictures showing P-selectin and PS exposure (ANX V binding) within thrombi formed in the absence (-Au) and in presence (+Au) of HAuCl_4_ (100 μM) are presented. In all pictures bars are 10 μm, *n* = 6, ^*^p < 0.05.

**Figure 3:**
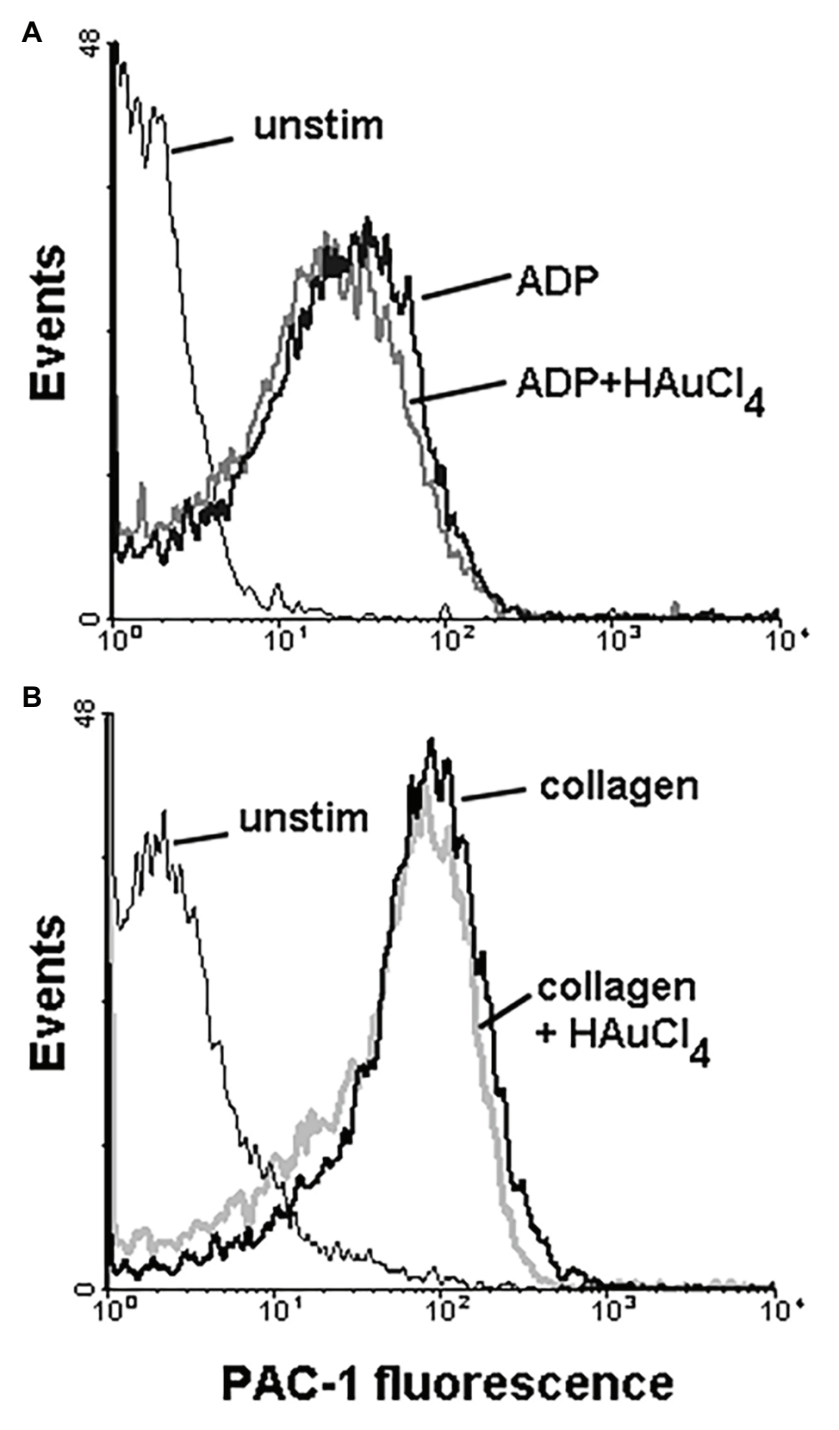
Effect of HAuCl_4_ on the binding of PAC-1 antibody to ADP‐ or collagen-stimulated platelets. Samples of PRP (2 × 10^8^ cells/ml), preincubated for 2 min without or with HAuCl_4_ (100 μM), were supplemented with PAC-1 antibody followed by the addition of ADP (10 μM) or collagen (15 μg/ml). After 5 min of incubation, samples were diluted by particle-free Tyrode buffer to quench antibody binding. PAC-1-related fluorescence of platelets after exposure to ADP **(A)** or collagen **(B)** were evaluated using flow cytometer. Unstimulated (unstim) platelets were prepared without HAuCl_4_. Result of one representative experiment (out of four) is presented.

**Figure 4:**
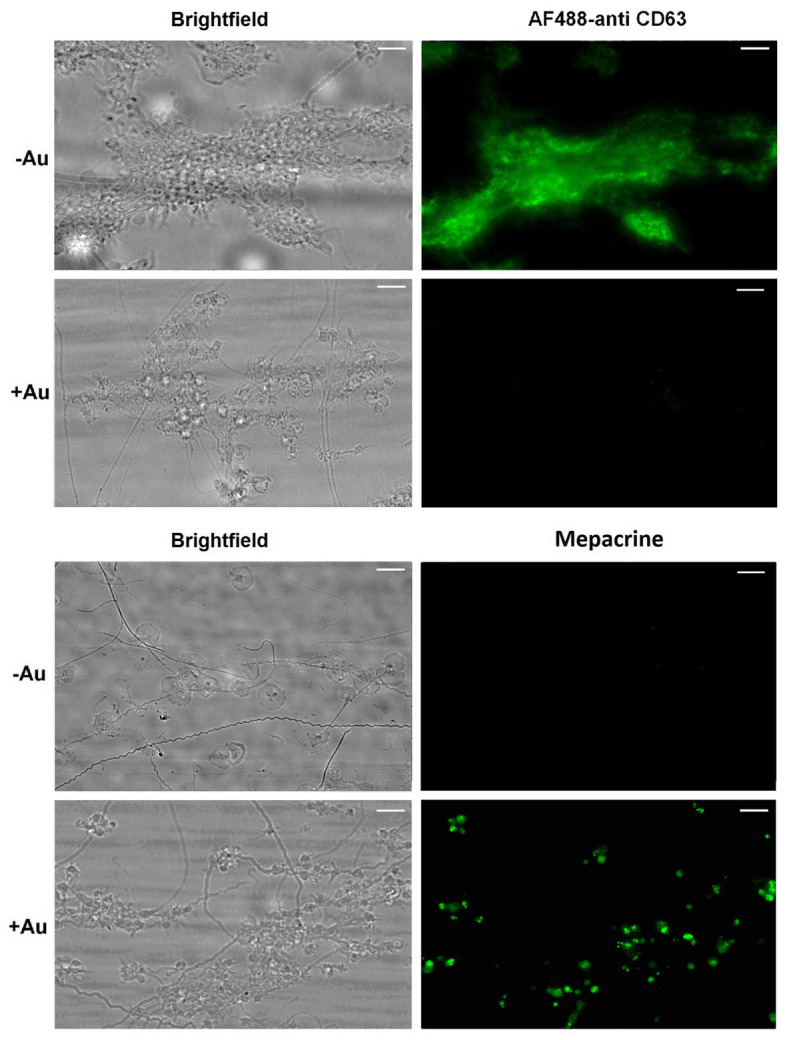
Inhibition of dense granules release from platelets under flow by HAuCl_4_. Platelet thrombi were formed under flow (1,000 s^−1^) on collagen followed by washing with buffer containing fluorescent anti-CD63 antibody to detect platelet plasma membrane-bound CD63 (marker of dense granules release). To additionally inspect retention of the dense granules content within platelets treated with HAuCl_4_ (+Au), platelets were loaded with mepacrine (which accumulates exclusively in dense granules). Resulted platelet monolayers developed under flow were compared with the monolayers composed of HAuCl_4_-untreated (-Au) platelets (obtained by the addition of tirofiban, a GPIIb/IIIa blocker) toward mepacrine retention. Representative pictures from one (out of three) experiment are presented. Blur effect on some pictures from experiments with Au(III) originates from different platelet size and their various orientation on collagen after adhesion due to fact that they do not spread in the presence of Au(III).

To check potential effect of Au(III) on thrombus formation and platelet activation status under flow conditions, we performed flow chamber assay under arterial shear rate (1,000 s^−1^) with concomitant visualization of platelet bound P-selectin (marker of secretion from alpha granules) and exposed PS (hallmark of platelet procoagulant response development). Treatment of whole blood samples with Au(III), at concentration previously shown to totally inhibit dense granules secretion in platelet suspension (Misztal et al., 2018) and strongly reducing morphological changes, i.e., 100 μM, resulted in a formation of mostly platelet monolayers on collagen-coated surfaces under arterial flow conditions without reducing surface area coverage by platelets (Figures 2A,B). After perfusion of ADP-treated samples over such monolayers, growth of thrombi emerged (Figure 2A). Au(III) diminished P-selectin exposure and ANX V binding (probe for exposed PS) to platelets adhered to collagen under flow (Figures 2A,C). Au(III) (100 μM) did not modulate PAC-1 antibody binding to GPIIb/IIIa receptors (PAC-1 binds to these receptors only in their active conformation) on ADP‐ or collagen-stimulated platelets (Figure 3), ruling out the possibility that Au(III)-related arrest of thrombi growth is a result of direct inhibition of GPIIb/IIIa activation. To additionally investigate whether formation of platelet monolayers on collagen under flow in the presence of Au(III) may be a result of attenuated secretion of secondary activators from platelet dense granules [but not a direct effect of Au(III) on aggregation], we compared: (1) the membrane exposure of CD63 – a marker of dense granules’ secretion – within thrombi incubated without or with Au(III) and (2) preservation of mepacrine [which accumulates exclusively within dense granules and is released outside platelet during secretion (Hanby et al., 2017)] in Au(III)-treated and untreated platelets. In case of “mepacrine preservation” experiments, control samples were supplemented with aggregation blocker – tirofiban, to obtain only platelet monolayers without blocking their ability to secretion. As can be seen, in case of Au(III)-treated platelets formation of monolayers was accompanied with the strong reduction of CD63 exposure (Figure 4, upper part) and with the retaining of mepacrine inside platelets (Figure 4, lower part).

### Inhibitory Effect of Au(III) on Platelet Procoagulant Response and Platelet-Fibrin Clot Formation

**Figure 5:**
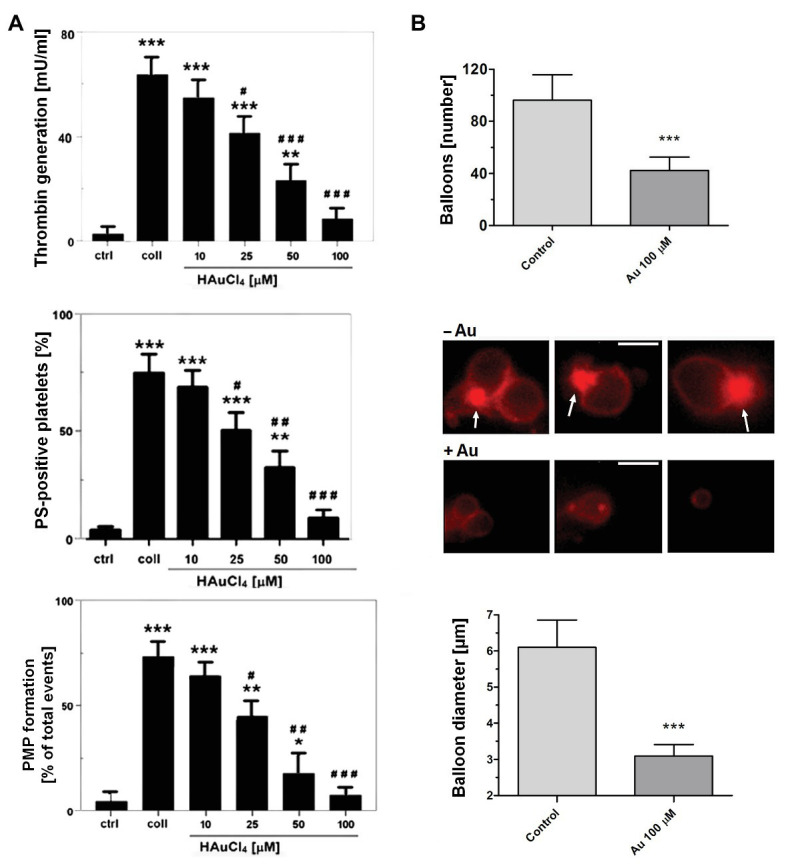
Reduction of platelet procoagulant response in the presence of HAuCl_4_. **(A)** To measure platelet-dependent thrombin generation, aliquots of washed platelets isolated from PRP samples treated or non-treated with HAuCl_4_, were preincubated for 2 min at 37°C in an aggregometer cuvette without stirring. Next, collagen (15 μg/ml) was added to each sample and, after an initial mixing (30 s), incubation was continued for 15 min without stirring. Phospholipid-dependent thrombin generation was evaluated using colorimetric assay. The amidolytic activity of thrombin was expressed in international units (U) per ml of platelet suspension. Control samples (ctrl) contained platelets without any additions. To measure surface PS exposure and platelet microparticles (PMP) formation, aliquots of PRP, preincubated for 2 min at 37°C without (ctrl) or with HAuCl_4_ (10–100 μM), were activated with collagen (15 μg/ml). After initial (30 s) stirring, suspensions were further incubated without stirring for 15 min. After incubation, samples were analyzed by flow cytometry. PS exposure in collagen-stimulated platelets is presented as percentages of PS (ANX V)-positive platelets. PMP were gated from a population of GPIIb(CD41a)-positive objects as those with small size (low forward scatter, FSC) and high PS expression [high fluorescein isothiocyanate (FITC)-ANX V signal]. Numbers of PMP are expressed as a percentage of total (10,000) gated objects. Mean values (±S.D.) of six independent experiments are shown. **(B)** To investigate effect of HAuCl_4_ on platelet ballooning, platelets (in whole blood) preincubated without (−Au) or with (+Au) HAuCl_4_ (100 μM) were allowed to adhere to a collagen-coated surface under shear rate of 1,000 s^−1^, followed by 4 min staining with Alexa Fluor 647-ANX V (1:200). After washing, platelets were imaged using fluorescent microscopy, and balloons diameters were determined at the plane of maximal balloon size. Arrows point platelet bodies with PS-rich “caps” distinctive from well-developed balloon-like structures. Bar is 5 μm. Images shown are representative for 126 platelets from five donors. In all panels: ^*^p < 0.05; ^**^p < 0.01; and ^***^p < 0.001 vs. control; ^#^*p* < 0.05; ^##^*p* < 0.01; and ^###^*p* < 0.001 vs. collagen.

**Figure 6:**
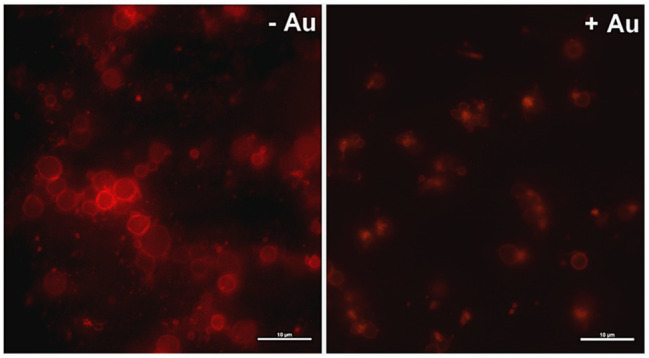
Effect of HAuCl_4_ on platelet ballooning. Samples of whole blood supplemented (+Au) or not (-Au) with HAuCl_4_ (100 μM) were perfused over collagen-coated surfaces to form thrombi. Balloon platelets, visualized with ANX V (red), were pictured from areas contained comparable surface coverage by thrombi using confocal microscope (×100/1.4 oil immersion objective). Bar is 10 μm, *n* = 5.

**Figure 7:**
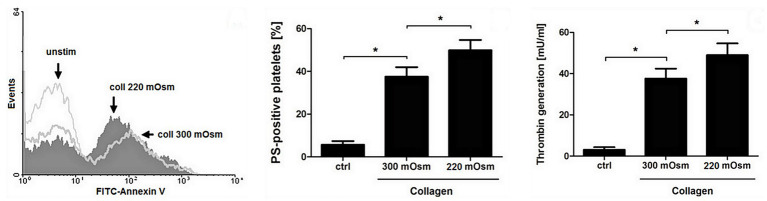
Osmolality-induced platelet swelling facilitates their procoagulant response. Washed platelets, suspended in a Tyrode buffer of osmolality of 300 or 220 mOsm/kg, were supplemented with a subthreshold concentration of collagen (8 μg/ml). After initial (30 s) stirring, suspensions were further incubated without stirring for 15 min at 37°C. After incubation, samples were analyzed for PS exposure, by means of flow cytometry (**left and middle panel**), and for platelet-dependent thrombin generation using amidolytic assay (**right panel**). PS exposure in collagen-stimulated platelets is presented as percentages of PS-positive platelets among 10,000 measured events. The data represent the mean values (±S.D.) from four independent experiments. Control (unstimulated) platelet samples (unstim, ctrl) were suspended in 300 mOsm/kg Tyrode buffer. Lower osmolality did not affect the PS-exposure in unstimulated platelets distinctly. In all panels: ^*^p < 0.05 vs. control.

**Figure 8:**
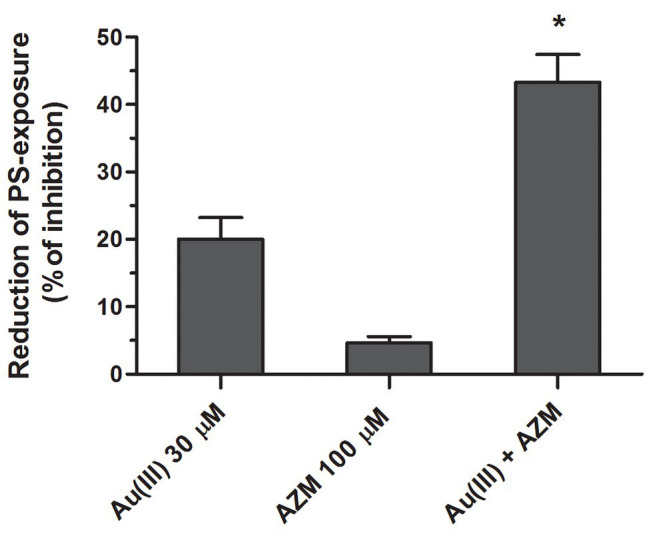
Synergistic reduction of PS exposure in collagen-stimulated platelets by HAuCl_4_ and acetazolamide. Samples of PRP, preincubated for 2 min at 37°C without (control samples) or with HAuCl_4_ [Au(III), 30 μM], acetazolamide (AZM, 100 μM), or combination of both compounds, were activated with collagen (15 μg/ml). After initial (30 s) stirring, suspensions were further incubated without stirring for 15 min and then analyzed by flow cytometry. Mean values (±S.D.) of three independent experiments are shown. ^*^p < 0.05 vs. Au(III) alone.

To obtain more detailed insight into reduction of platelet procoagulant response by Au(III), we investigated its effect on platelets PS exposure and microparticles shedding by flow cytometry, platelet ballooning on collagen-coated surfaces, and platelet-dependent thrombin generation. As can be seen, platelet-dependent thrombin formation, PS exposure, and the shedding of PMPs in the fluid phase, all evoked by collagen, were diminished by Au(III) in a dose-dependent manner (25–100 μM; Figure 5A). The number and size of balloon-like structures and formation of PS-rich “caps” in collagen-adhered platelets were markedly reduced by 100 μM of Au(III) (Figures 5B, 6) i.e., by a concentration previously shown to strongly inhibit collagen-evoked water uptake by human platelets (Misztal et al., 2018). PS exposure (Figure 7, left and middle panel) and platelet-dependent thrombin generation (Figure 7, right panel) in collagen-stimulated platelets were distinctly higher under hypotonic conditions, which predispose platelets to inflate. Incubation of PRP samples with submaximal Au(III) (30 μM) and acetazolamide (100 μM), a carbonic anhydrase inhibitor shown to strongly inhibit AQP1 (Gao et al., 2006), resulted in a synergistic reduction of PS-exposed platelet population after stimulation by collagen (Figure 8).

**Figure 9:**
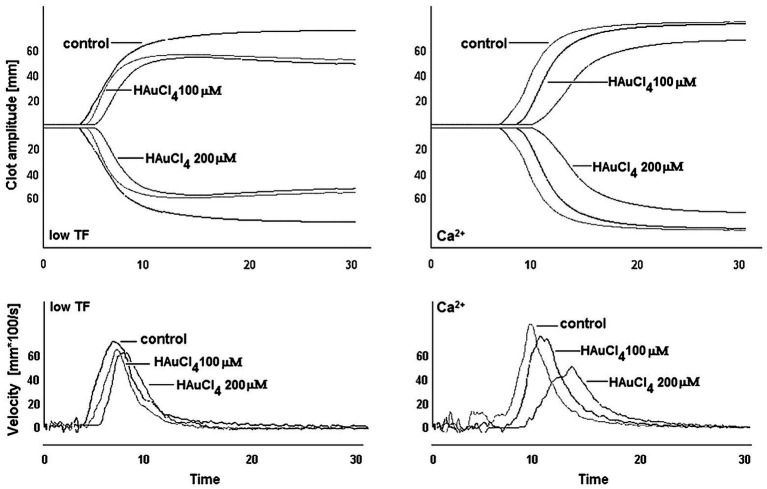
Effect of HAuCl_4_ on the kinetics of clot formation in whole blood. Samples of whole blood were preincubated for 2 min at 37°C with HAuCl_4_ (0–200 μM). After that, 320 μl of samples were transferred to thromboelastometer cups and gently mixed with 20 μl of TF/calcium chloride mixture (final concentrations in sample: 140 ng/ml of TF and 12 mM CaCl_2_ – “low TF”) or with CaCl_2_ (“Ca^2+^”, 12 mM final concentration). Representative thromboelastogram profiles and derivatives (showing clot formation velocity) for selected HAuCl_4_ additions are presented (one out of six independent experiments).

To investigate whether Au(III)-evoked diminishing of platelet procoagulant response may affect the kinetics of platelet-fibrin clot formation, we measured whole blood clot development by thromboelastometry. Au(III) (25–200 μM) affects the kinetics of clot formation, triggered by both tissue factor (“low TF”) or calcium (“Ca^2+^”), dose-dependently (Figure 9). Treatment of whole blood with 200 μM of Au(III) resulted in a prolongation of CT by ~100% (TF) or ~50% (calcium) and in a significant reduction of alpha angle and clot formation rate values (Table 1) – parameters describing dynamics of clot propagation (Sorensen and Ingerslev, 2004). Au(III), up to 200 μM, did not affect coagulation kinetics in platelet-depleted plasma (not shown).

**Table 1:** Kinetics of clot formation in whole blood in the presence of chloroauric(III) acid (HAuCl_4_).


Addition	CT (s)	alpha (angles)	MaxVel (mm × 100/s)	t-MaxVel (s)	MCF (mm)	AUC (mm × 100)
A	None (control)	186 ± 43	78 ± 2	29 ± 4	297 ± 65	78 ± 3	8,283 ± 1,441
	HAuCl_4_ 10 μM	199 ± 52	78 ± 3	28 ± 3	317 ± 59	77 ± 3	8,177 ± 1,069
	HAuCl_4_ 25 μM	235 ± 39^*^	73 ± 4	26 ± 4	348 ± 62^*^	74 ± 3	7,792 ± 1,082
	HAuCl_4_ 50 μM	279 ± 61^**^	69 ± 2^**^	21 ± 4^**^	400 ± 55^**^	69 ± 3^**^	7,021 ± 947^*^
	HAuCl_4_ 100 μM	314 ± 45^***^	65 ± 2^**^	17 ± 3^***^	498 ± 71^***^	60 ± 4^**^	6,462 ± 1,320^**^
B	None (control)	389 ± 52	76 ± 3	26 ± 4	514 ± 55	76 ± 4	7,799 ± 1,232
	HAuCl_4_ 10 μM	429 ± 46	74 ± 3	25 ± 3	546 ± 73	73 ± 3	7,296 ± 954
	HAuCl_4_ 25 μM	492 ± 33^**^	71 ± 2^*^	21 ± 3^*^	613 ± 76^*^	68 ± 3^*^	6,874 ± 1,146^*^
	HAuCl_4_ 50 μM	541 ± 44^**^	67 ± 3^**^	16 ± 4^**^	651 ± 67^**^	63 ± 3^**^	6,758 ± 1,054^*^
	HAuCl_4_ 100 μM	607 ± 65^***^	63 ± 2^**^	13 ± 3^***^	715 ± 57^***^	59 ± 4^**^	6,320 ± 1,284^**^

Aliquots (1 ml) of whole blood were incubated in polypropylene tubes at 37°C for 2 min without (control) or with HAuCl_4_ added to the final concentrations as indicated. Next, 320 μl of sample was transferred to cup containing with 20 μl of activation mixture. Coagulation was triggered by a mixture of tissue factor (TF) and calcium chloride (140 ng/ml and 12 mM final concentrations, respectively); (A) or by recalcification (12 mM final concentration of calcium chloride); (B). Further details in Materials and Methods. The number are means ± S.D. of six independent experiments performed on a single platelet preparation, each in triplicate.

* *p* < 0.05;

** *p* < 0.01;

*** *p* < 0.001 vs. control.

### Au(III)-Related Reduction of the Presence of PS-Exposing Cells in FeCl_3_-Induced Thrombosis in Mice

**Figure 10:**
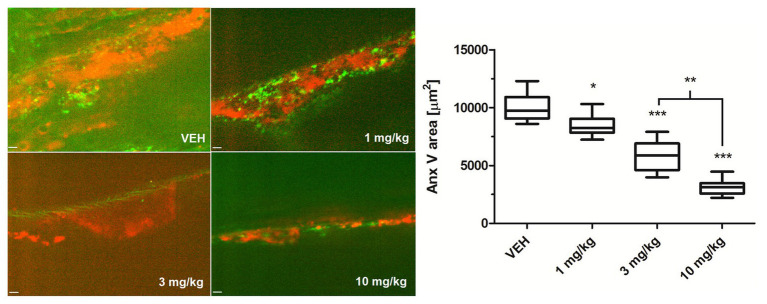
The effect of HAuCl_4_ on thrombus-related PS exposure in ferric chloride (III) (FeCl_3_)-induced thrombosis in wild-type mice. The areas of Anx V-labeled platelets during thrombus formation in mice treated acutely with vehiculum (saline, VEH), or HAuCl_4_ (at doses of 1, 3, or 10 mg/kg for 2 min prior to the exposure to FeCl_3_) were quantified by scanning confocal microscope. Representative confocal images of the distribution of Anx V-positive cells (red color) in a thrombus 2 min after FeCl_3_-induced injury are presented (objective 20×, vessel diameter ~150 μm). Anx V-negative platelets and vessel wall are in green. The visible background signals in the pictures result from the fact that confocal microscopy system adjusts the contrast and the background fluorescence automatically to obtain the most sharp picture of studied objects. Data are presented as mean ± SEM. ^*^p < 0.05; ^**^p < 0.01; and ^***^p < 0.001 vs. VEH. *n* = 9.

To examine whether Au(III) may modulate thrombosis *in vivo*, we measured area of PS-exposing cells within thrombus evoked by a short exposure of murine mesenteric vein to FeCl_3_, assessed by intravital microscopy. As is seen in Figure 10, intravenous administration of Au(III) (1–10 mg/kg) resulted in a substantial reduction in the content of PS-exposing cells.

### Lack of Impact of Au(III) on Calcium Signal in Stimulated Platelets

**Figure 11:**
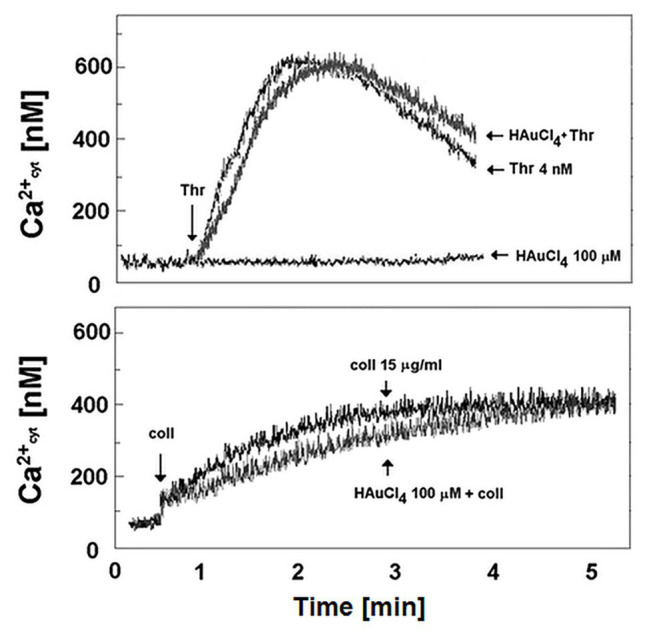
Effect of HAuCl_4_ on thrombin‐ and collagen-evoked calcium signal in washed platelets. Fura-2-loaded washed platelets were preincubated for 2 min without or with HAuCl_4_ (0–100 μM) at 37°C. After that, 35 μl of platelets suspension were transferred into thermostated (37°C) cuvette containing 1965 μl of Tyrode buffer supplemented with CaCl_2_ (1 mM). Thrombin (4 nM) or collagen (15 μg/ml) was added to the stirred suspension (pointed by the arrow on the left side of each image) after stabilization of basal fluorescent signal. Representative results from one experiment (out of six) is presented.

To verify if Au(III)-related reduction of platelet PS exposure might be a result of diminished calcium signal, we recorded rises in cytosolic calcium concentration following treatment of stirred washed platelets by thrombin or collagen in the presence and absence of Au(III). Au(III) (100 μM) did not moderate a rise in cytosolic calcium concentration following platelet activation (Figure 11).

### Insignificant Effect of Au(III) on Platelet-Mediated Clot Retraction

**Figure 12:**
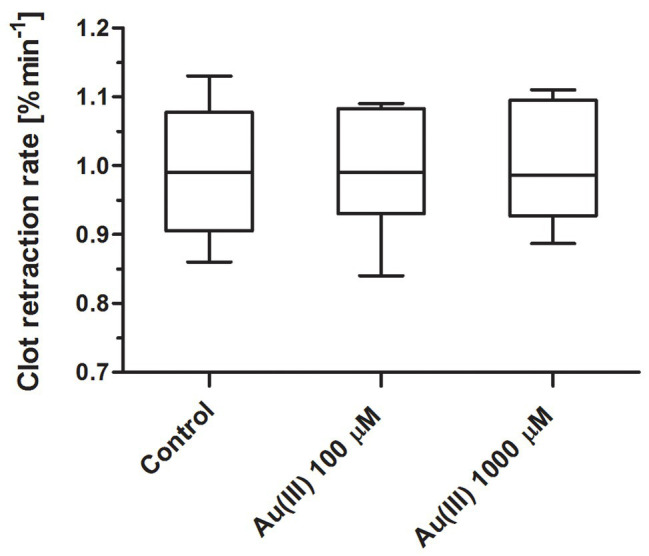
Lack of distinct effect of HAuCl_4_ on clot retraction rate. Whole blood samples were incubated without (control) or with HAuCl_4_ at final concentrations as indicated. Clotting was triggered by recalcification. Clot surface areas were assessed by digital processing, and the clot retraction rates were calculated from the slope of the clot volume against time (min^−1^). Data are presented as median (with maximum and minimum values) from six independent experiments, each in triplicate.

To test whether Au(III) may exert its effect on platelets *via* inhibition of thioredoxin reductase (TrxR) or impairment of mitochondria, we recorded the rate of platelet-fibrin clot retraction – a phenomenon known to be sensitive to both TrxR inhibition (Metcalfe et al., 2016) and attenuation of mitochondrial energy production (Misztal et al., 2014, 2015). As we shown in Figure 12, Au(III) (up 1,000 μM) did not decrease the rate of clot retraction measured in whole blood.

## Discussion

### Role of AQPs in Platelet Spreading and Filopodia Formation

Here we demonstrate directly that Au(III) reduces formation of lamellipodia and filopodia in activated platelets (Figures 1A,B).

The current paradigm of filopodia and lamellipodia formation considers the parallel involvement of both local osmotic disequilibrium, at the site of the protruding plasma membrane produced by local ion fluxes, and the subsequent rapid water influx supported by AQPs (Mattila and Lappalainen, 2008; Schwab et al., 2012). Resulted plasma membrane protrusions are further filled by elongating actin microfilaments and microtubules (Patel-Hett et al., 2008; Li et al., 2010; Aslan et al., 2013). Previously, we excluded the modulatory effect of Au(III) (at concentrations shown here to strongly reduce filopodia and lamellipodia formation) on the influx of osmotically active Cl^−^ and Na^+^ ions into the platelet cytosol (Misztal et al., 2018). Since, as we show here, these Au(III) concentrations did not modulate the rise in microtubules and F-actin filaments content following activation (Figure 1C), one can hypothesize that in activated platelets AQPs contribute to filopodia and lamellipodia formation by driving the elongation of growing protrusion but they are not directly linked with the filaments polymerization process. Similar mechanism is considered in case of migrating cells, where AQPs deficiencies have been found to be associated with impaired cell motility due to inhibition of filopodia and lamellipodia formation (Papadopoulos et al., 2008).

### Involvement of AQPs in Platelet Activation and Thrombus Formation Under Flow

Here we show that under arterial flow conditions, Au(III) strongly attenuated growth of thrombi on collagen, without visible effect on the coverage of surface by platelets, i.e., the adhesion (Figures 2A,B). Since the thrombi growth emerged after perfusion of ADP-pretreated samples, this suggests that: (1) Au(III) does not interfere with interactions between platelet receptors and collagen and (2) that activation of GPIIb/IIIa receptors (necessary for platelet aggregation and thrombi growth) is not inhibited by Au(III) directly. That second idea was further confirmed by observation that Au(III) did not modulate PAC-1 binding to activated platelets (Figure 3).

These results suggest that a possible cause of thrombi growth arrest in the presence of Au(III) might be reduction of platelet secretion. This hypothesis is supported by the following observations: (1) supplementation of samples with exogenous ADP restored normal thrombi development under flow (Figure 2A); (2) Au(III) notably reduced the exposure of platelet membrane-bound P-selectin and CD63, markers of secretion from alpha and dense granules, respectively (Figures 2C, 4); and (3) incubation of whole blood samples with Au(III) resulted in a preservation of mepacrine within dense granules of platelets accumulated on collagen under flow (Figure 4).

The possibility that thrombus formation may be attenuated by Au(III)-related inhibition of thromboxane A_2_, (TxA_2_, a vital secondary platelet agonist) formation is also unlikely since, in our previous study, we did not observe diminishing of arachidonic acid-induced platelet aggregation (which is directly TxA_2_-dependent) in the presence of Au(III) (Misztal et al., 2018).

These observations indicate that platelet AQPs are involved in a process of thrombus formation under flow conditions *via* their contribution to platelet secretion.

### Engagement of AQPs in Platelet Procoagulant Response

The contact of platelets with the subendothelial matrix at the site of an injury initiates the rapid transformation of these cells into balloon-like structures, which is further associated with a formation of PMPs (Agbani et al., 2015, 2018; Mattheij et al., 2016). The above mentioned processes are associated with a distending of plasma membrane and exposure of PS, which support the local generation of thrombin (Heemskerk et al., 2013; Agbani et al., 2015; Mattheij et al., 2016).

Since ballooning and PMP shedding are relatively fast processes, here we hypothesize that the contribution of AQPs may be required for supporting a rapid rise of hydrostatic pressure in the platelet cell body and for localization of a site of membrane balloon protrusion. This hypothesis is supported by the observation that under hypotonic conditions, where platelets were further predisposed to water uptake, we observed the augmented exposure of PS and thrombin generation associated with it (Figure 7).

Our results indicate that Au(III) diminished broad spectrum of platelet procoagulant response, both in fluid phase and on collagen-coated surfaces, i.e., PS exposure (Figures 2C, 5A), ballooning (Figures 5B, 6), and PMP formation (Figure 5A), and platelet-dependent thrombin generation (Figure 5A). Accordingly, Au(III) prolonged CT, reduced the maximum velocity of clot formation and alpha angle (Figure 9; Table 1) measured by thromboelastometry. Since all the above mentioned parameters are known to be well-correlated with the rate of thrombin formation (Sorensen and Ingerslev, 2004), these findings point to the involvement of AQPs in a platelet-dependent thrombin generation and clot formation. It is supported by the observation that Au(III) collaborates with acetazolamide (AQP1 inhibitor; Gao et al., 2006) in a potent, synergistic reduction of PS exposure on collagen-stimulating platelets (Figure 8). Since PS exposure is augmented by secondary platelet stimulators, the most importantly by secreted ADP (Dorsam et al., 2004; van der Meijden et al., 2005), inhibition of platelet secretion, observed in the presence of Au(III), may thus act in concert with a diminishing of AQP-mediated water influx into platelet cytosol resulting in a net reduction of PS exposure.

Of importance, reduction of platelet PS exposure was also observed *in vivo* in FeCl_3_-induced mouse model of thrombosis, where Au(III) decreased the area of ANX V-positive cells within thrombus in a dose-dependent manner (Figure 10). It is worth to emphasize that the doses of Au(III) administered during *in vivo* part of our study (1–10 mg/kg) correspond to the concentration range of approximately 40–400 μM (calculations based on the weight of animals used and predicted amount of circulating blood). These Au(III) concentrations have been previously found to completely block platelet secretion without producing disturbances in erythrocytes’ membranes in whole human blood (Misztal et al., 2018), reported by other authors in red blood cells suspended in artificial media (Suwalsky et al., 2004). It is worth to emphasize that in this experimental model, the presence of erythrocytes within thrombi is expected (Ciciliano et al., 2015). Taking into consideration that erythrocytes express at least few AQP isoforms (Roudier et al., 1998; Agre et al., 2002) and can expose PS *in vivo* (Weisel and Litvinov, 2019), it is possible that observed here Au(III)-mediated reduction of thrombi-related PS exposure is attributed both to platelets and erythrocyte, however, the engagement of endothelial cells in exposing of PS in this model cannot be ruled out. Intriguingly, treatment of platelets with Au(III) did not affect the generation of a calcium signal in thrombin‐ or collagen-activated platelets notably (Figure 11), a well-accepted prerequisite for the development of platelet procoagulant response (Varga-Szabo et al., 2009; Obydennyy et al., 2016). Our observation is contrary to those obtained by Agbani et al. (2018), where authors observed diminished calcium signal in platelets from AQP1-null mice adhered to collagen. Possible explanation of this inconsistency is the variation of experimental systems – Agbani et al. (2018) measured calcium signal in single platelets adhered to a solid thrombotic surface, while our system comprised washed platelets stimulated in a stirred suspension where both, the inhibitor and the agonists could be much more dispersed.

To sum up, AQPs are likely to be associated with PS exposure and platelet-related amplification of blood coagulation. The presence of the AQPs’ blocker in blood vessels contributes to decreasing of thrombosis *in vivo*.

### Clinical Implications and Limitations

Recently, much attention has been directed to antitumor activity of Au(III)-containing compounds, which are believed to exert their effect *via* inhibition of TrxR and impairment of mitochondria (Barnard and Berners-Price, 2007; Bertrand et al., 2018). In case of platelet, however, Au(III) at concentrations even 10 times higher than those notably inhibiting secretion, thrombus formation, and procoagulant response was unable to alter rate of clot retraction (Figure 12), previously shown to be mitochondria-dependent and sensitive to TrxR inhibition (Misztal et al., 2014, 2015; Metcalfe et al., 2016). Taken together, we conclude that observed here impact of Au(III) on platelet responses is, at least substantially, associated with its effect on platelet AQPs.

It has been proved that the most widely used antiplatelet drug – aspirin, lost its antithrombotic efficacy at a high, pathological shear rates, which was observed both *in vitro* and in patients with severe atherosclerotic lesions (Veen et al., 1993; Li et al., 2014). Due to this fact, dual antiplatelet therapy is often employed, comprising using of aspirin in combination with ADP receptor (P2Y12) blockers in heart attacks and following the placement of a coronary artery stent (Yusuf et al., 2001; Steinhubl et al., 2002). Currently, clopidogrel (P2Y12 antagonist) at the optimal clinical dose appears as the most efficient, shear-independent inhibitor of arterial thrombus formation (Roald et al., 1994; Yusuf et al., 2003). However, according to clinical data (U.S. Food and Drug Administration, 2010) about 14% of U.S. patients are poor metabolizers of clopidogrel (which requires bioactivation by the liver CYP2C19 enzyme), which results in a low plasma concentration of an active form of the drug and hence in a significant reduction of its beneficial properties. Attenuation of ADP releases from activated platelets by AQPs inhibitors may be therefore potentially effective antiplatelet strategy for clopidogrel poor metabolizers.

Additional studies, including further *in vivo* investigations and assessing of shear-dependence are required to better evaluate antiplatelet capacity of AQPs inhibitors. Furthermore, the specific engagement of particular isoforms of AQPs in platelet physiology [beyond already investigated AQP1 (Agbani et al., 2018) and, partially, AQP7 (Goubau et al., 2013)] – which was beyond the scope of this study – should be investigated in future.

### Conclusion

In conclusion, this study suggests that, in human platelets, APQs localized in secretory granules play a crucial role in platelet secretion and hence in thrombus formation under flow conditions. Rapid water transport through AQPs localized in platelet plasma membrane seem to be crucial in filopodia and lamellipodia formation process and is likely to participate in membrane ballooning and PS exposure. Reduction of platelet PS exposure observed *in vitro* (in human blood) and *in vivo* (in murine blood) along with the results of thromboelastometric analyses suggests antithrombotic benefits of the inhibition of platelet AQPs. Our findings establish basis for future studies with AQPs inhibitors (including isoform specific ones), which may be used as active compounds in drug delivery systems designed to be platelet-specific. Use of materials that could locally release platelet-targeted AQPs inhibitors from eluting stents and blood vessels prosthetics under elevated shear rate – and thus minimize thrombus formation on artificial surface – might be another potential application for AQP inhibitors as antiplatelet agents.

## Data Availability Statement

The raw data supporting the conclusions of this article will be made available by the authors, without undue reservation.

## Ethics Statement

The studies involving human participants were reviewed and approved by Bioethics Committee at the Medical University of Bialystok. The patients/participants provided their written informed consent to participate in this study. The animal study was reviewed and approved by Local Ethical Committee on Animal Testing at the Medical University of Bialystok.

## Author Contributions

TM designed research, performed experiments, analyzed the data, and wrote the manuscript. AG performed experiments, analyzed the data, and wrote the manuscript. JB-J and NM performed experiments and analyzed the data. EC and TR analyzed the data. MT wrote the manuscript. All authors contributed to the article and approved the submitted version.

### Conflict of Interest

The authors declare that the research was conducted in the absence of any commercial or financial relationships that could be construed as a potential conflict of interest.

## Footnotes

^1^http://imagej.nih.gov/ij/

## Abbreviations

AQPs: Aquaporins
ADP: Adenosine diphosphate
PS: Phosphatidylserine
NPA: Asparagine-proline-alanine
Cys: Cysteine
AF: Alexa fluor
FITC: Fluorescein isothiocyanate
ROTEM: Rotational thromboelastometry
DMSO: Dimethyl sulfoxide
PBS: Phosphate-buffered saline
TF: Tissue factor
PE: Phycoerythrin.
